# Retrospective analysis of secondary enucleation for uveal melanoma after plaque radiotherapy

**DOI:** 10.1186/s12886-022-02387-x

**Published:** 2022-04-09

**Authors:** Heng Wang, Ruiheng Zhang, Yining Wang, Rongtian Chen, Yueming Liu, Yang Li, Wenbin Wei

**Affiliations:** Beijing Tongren Eye Center, Beijing Key Laboratory of Intraocular Tumor Diagnosis and Treatment, Beijing Ophthalmology and Visual Sciences Key Lab, Medical Artificial Intelligence Research and Verification Key Laboratory of the Ministry of Industry and Information Technology, Beijing Tongren Hospital, Capital Medical University, Beijing, 100730 China

**Keywords:** Uveal neoplasms, Melanoma, Radiotherapy, Enucleation

## Abstract

**Background:**

Uveal melanoma (UM) is the most common primary intraocular malignancy in adults. Plaque brachytherapy (PRT) is widely accepted as an effective globe-conserving treatment modality for UM. However, local treatment failure and complications lead to the enucleation of irradiated eyes. We conducted this study to explore the causes and long-term prognosis for UM patients who accepted secondary enucleation after plaque radiotherapy.

**Methods:**

This was a retrospective cohort study. Data of patients who underwent secondary enucleation for UM after plaque radiotherapy, from July 2007 to July 2019, at Beijing Tongren Hospital were analyzed. Kaplan–Meier analysis was performed to assess the probability of indications, metastasis, and metastasis-related death. Cox regression analysis was used to analyze associations of the prognostic factors.

**Results:**

Eight hundred and eighty patients were clinically diagnosed with uveal melanoma and initially treated by iodine-125 plaque radiotherapy, 132 of whom underwent secondary enucleation and pathological examination in the same hospital. Fifty-two (39.4%) eyes were enucleated simply because of uncontrollable neovascular glaucoma (NVG). Forty-four (33.3%) patients suffered from tumor recurrence. Tumor non-response occurred in 18 (13.6%) cases. Ten (7.6%) eyes received enucleation entirely due to other types of glaucoma. Failure to preserve the eyes for other reasons occurred in eight (6.1%) patients. At a median follow-up of 58.1 [IQR: 40.9–90.5] months, the systemic spread was detected in 45 (34.1%) patients, and 38 of them died. On multivariate analysis, tumor largest basal diameter (HR 1.15 [95% CI: 1.01, 1.31]), tumor non-response (HR 7.22 [95% CI: 2.63, 19.82]), and recurrence (HR 3.29 [95% CI: 1.54, 7.07]) were risk factors for metastasis. Increased age (HR 1.54 [95% CI: 1.07, 2.23]), tumor non-response (HR 7.91 [95% CI: 2.79, 22.48]), and recurrence (HR 3.08 [95% CI: 1.13, 7.23]) were risk factors for metastasis-related death.

**Conclusions:**

NVG was the major reason for secondary enucleation for Chinese UM patients after PRT. Tumor non-response and recurrence were associated with a significantly higher risk of long-term metastasis and metastasis-related death.

## Strengths and limitations of this study

- This study firstly investigated the reasons for enucleation in East Asian uveal melanomas after brachytherapy.
- Uncontrollable neovascular glaucoma was the primary cause of secondary enucleation for Chinese uveal melanoma patients.
- Major limitations included the nature of the retrospective study, the exclusion of radiation-sensitive cases, and the unavailability of molecular genetic analysis.
- There was a single-center bias as we excluded eyes enucleated in other hospitals.

## Introduction

Uveal melanoma (UM) is the most common primary intraocular malignancy in adults, with an incidence rate of 0.42 and 0.64 per million per year in South Korea and Japan [1, 2]. The treatments for UM can be divided into two categories: enucleation and global salvage treatment. Brachytherapy is an effective globe-conserving treatment modality for UM worldwide, whereas other treatments such as transpupillary thermotherapy (TTT), proton beam, charged particle radiotherapy, photodynamic therapy, and tumor resection are additionally available [3].

Although plaque radiotherapy cannot decrease metastasis or mortality rate [4], it can effectively control tumors, preserve eyes and retain visual acuity [5, 6]. Miguel et al. pointed out that the cumulative probabilities of globe preservation after PRT by Kaplan–Meier analysis at 3, 5, 10, and 15 years were 93%, 88%, 81%, and 73%, respectively [7]. Successful clinical regression of the tumor was observed in most cases, but occasionally, local radiation failure and complications led to enucleation of irradiated eyes. Thus, it is necessary to study the indications for secondary enucleation after PRT. However, it is worth noting that most studies of this area were conducted based on the Caucasian population [8, 9], while evidence on East Asians is still scarce. Considering the ethnic disparities of UM in clinical characteristics, progression, and long-term prognosis, the purpose of this study was to explore the causes and long-term prognosis of secondary enucleation for East Asian UM patients who received PRT.

## Methods

### Patients

This was a retrospective cohort study. We collected clinical data from July 2007 to July 2019 at Beijing Tongren Hospital. Eight hundred and eighty patients were clinically diagnosed with uveal melanoma and initially treated by iodine-125 (I^125^) plaque radiotherapy. A total of 132 patients who underwent enucleation after PRT and pathological examination in our center were analyzed. This study adhered to the Declaration of Helsinki and was approved by the Medical Ethics Committee of Beijing Tongren Hospital, Capital Medical University. Written informed consent was obtained from all participants.

### Methods

#### Clinical Data

The following clinical data were obtained: gender, age, ocular and systemic medical history, laterality, best-corrected visual acuity (BCVA), intraocular pressure (IOP), tumor shape, tumor orientation, ciliary body involvement, tumor largest basal diameter (LBD), tumor thickness, AJCC classification (8th Edition) [10, 11], location of anterior and posterior margin, extraocular and disc invasion, vitreous hemorrhage, and subretinal fluid. Pathological types and additional treatments were noted.

Color Doppler imaging (CDI) was used for tumor LBD and thickness. The shape (mushroom-like, flat, dome, diffuse, and irregular), tumor orientation (nasal, nasal-superior, superior, temporal-superior, temporal, temporal inferior, inferior, nasal-inferior, macular, and multiple lesions centered by disc), tumor anterior and posterior margin (iris, ciliary body, ora serrata to equator, equator to macula, and macula), disc involvement, and ciliary body involvement were determined by magnetic resonance imaging (MRI), CDI, slit-lamp biomicroscopy, indirect ophthalmoscopy, and fundus image. MRI and CDI were used to detect extraocular invasion. Indirect ophthalmoscopy, optical coherence tomography, and CDI were used to evaluate subretinal fluid and vitreous hemorrhage. According to Callender classification [12], tumor pathological types were divided into epithelial cell type, spindle cell type, mixed cell type, necrotic type, and others. The pathological types were evaluated by senior pathologists in Beijing Tongren Hospital.

#### PRT surgery data

I^125^ brachytherapy was applied to medium-sized (2.5–10 mm in apical height and ≤ 16 mm in LBD) [13]tumors and large tumors (> 10 mm in apical height or > 16 mm in LBD) [13, 14] that refused enucleation. The dose to be delivered to the tumor apex was 100 Gy. The plaque was 4 mm larger than tumor LBD. The radiation time was decided based on tumor height [15]. The anterior margin of the tumor base was determined by indirect ophthalmoscopy, and I^125^ plaque was implanted on the sclera during the operation. All the surgeries were performed by the same surgeon.

#### Follow-up

Patients were reviewed one month, three months, six months, and one year after plaque removal. Regular semi-annual to annual follow-up was required. The follow-up time was recorded started from the initial treatment.

The abdominal ultrasonography, orbital MRI, chest computed tomography (CT), and laboratory test for liver function were performed every visit beyond ocular examination. Positron emission tomography-computed tomography (PET-CT) was performed when the metastatic spread was suspected. All examinations were performed by the same methods.

The outcomes were recorded and included time from plaque implantation to enucleation, melanoma metastasis, and death from melanoma or other reason. Reasons for enucleation were noted. Tumor recurrence was considered when the tumor shrunk after treatments but subsequently increased by more than 0.5 mm in basal diameter or a 15% increase in height. Tumor non-response was defined as tumor no regression and growth within the first year after PRT. Neovascular glaucoma was defined as the presence of IOP ≥ 21 mmHg on a minimum of three occasions with iris neovascularization. Other types of glaucoma were defined as the presence of IOP ≥ 21 mmHg on a minimum of three occasions without iris neovascularization. Scleral necrosis was defined as scleral thinning and increased transparency without tumor enlargement.

#### Statistical analysis

Categorical variables were described using frequencies and percentages, while numerical variables were summarized as median, mean, standard deviation, and interquartile interval (IQR). Kaplan–Meier analysis was performed to estimate the cumulative probability of indications, metastasis, and death. Univariate and multivariate Cox proportional hazards regression analysis was used for the presumed risk factors for metastasis and metastasis-related death separately. *P*-value < 0.05 was considered for statistical significance. All analyses were performed in Stata (15.0, StataCorp LLC, College Station, TX, USA).

## Results

### Clinical features and follow-up

A total of 132 patients received secondary enucleation and pathological examination for UM in Beijing Tongren Hospital. Patients’ demographic and clinical features at presentation were summarized in Table 1. The median follow-up was 58.1 (range 7.0–154.0, IQR: 40.9–90.5) months. The median time from primary PRT to secondary enucleation was 25.0 (range 0.5–139.3, IQR: 11.0–38.9) months.

**Table 1:** Baseline clinical information of secondary enucleation patients (*n* = 132)

Characteristic	Value
**Age, years**	
Median (mean, range)	47 (45.5,17–79)
**Gender, No. (%)**	
Male	61 (46.2%)
Female	71 (53.8%)
**Laterality, No. (%)**	
Right	71 (53.8%)
Left	61 (46.2%)
**Tumor shape, No**	
Mushroom-like	45
Flat	4
Hemisphere	66
Irregular	15
Diffuse	2
**Tumor Location, No**	
Superior	10
Nasal	12
Inferior	8
Temporal	21
Temporal-superior	26
Nasal-superior	12
Nasal-inferior	10
Temporal inferior	23
Macula	8
Multiple lesions	2
**Tumor dimensions, mm**	**Median [Interquartile Range]**
Tumor Thickness	6.9 [5.2–9.4]
Tumor Largest Basal Diameter	12.8 [10.6–14.8]
**AJCC Stages, No**	
IA	7
IIA	42
IIB	64
IIIA	15
IIIB	4
**Tumor Anterior Margin, No**	
Iris	1
Ciliary Body	18
Ora Serrata to Equator	53
Equator to Macula	58
Unclear	2
**Tumor Posterior Margin, No**	
Ora Serrata to Equator	4
Equator to Macula	54
Macula	72
Unclear	2
**Ciliary body involved, No. (%)**	19 (14.4%)
**Disc involved, No. (%)**	22 (16.2%)
**Extraocular extension, No. (%)**	2 (1.5%)
**Subretinal Fluid, No. (%)**	121(91.7%)
**Vitreous Hemorrhage, No. (%)**	6 (4.4%)

### Indications for secondary enucleations after PRT

Neovascular glaucoma was the prime cause of enucleation after PRT, followed by tumor recurrence, tumor non-response, and other types of glaucoma (Table 2).

**Table 2:** Indications for secondary enucleation

Indications	Number	Percentage
Neovascular Glaucoma	52	39.4%
Recurrence	44	33.3%
Tumor Non-response	18	13.6%
Other Types of Glaucoma	10	7.6%
Other Reasons	8	6.1%
Total	132	100%

**Fig. 1:**
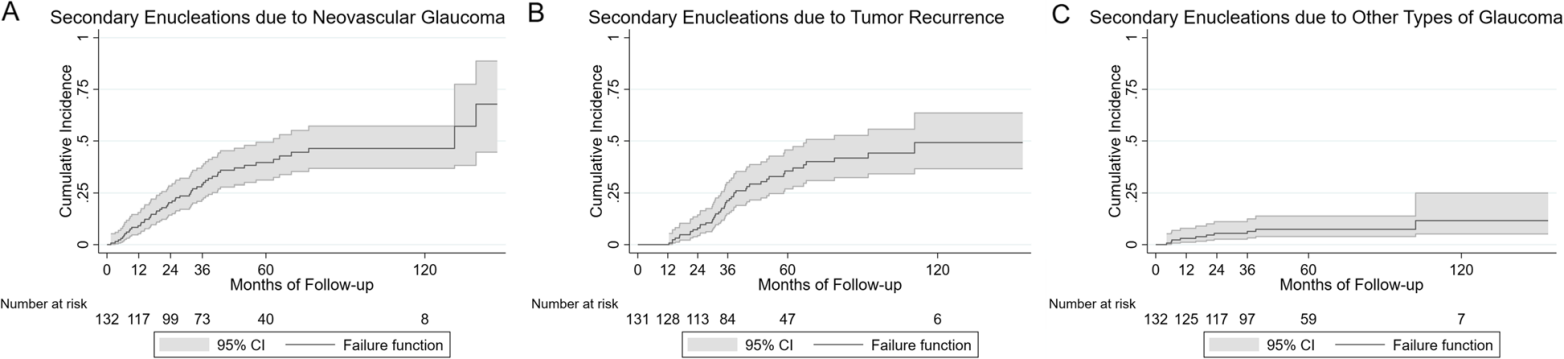
Kaplan–Meier analysis for secondary enucleations due to NVG (A), tumor recurrence (B), and other types of glaucoma (C) with 95% confidence intervals

Fifty-two (39.4%) eyes were enucleated simply because of NVG. The median time to enucleation was 23.9 months (range: 1.5–139.3 months, IQR: 12.8–39.0 months) (Fig. 1). Patients received standard treatments, including IOP lowering medications, intravitreal anti-vascular endothelial growth factor (VEGF) treatment (10 eyes), and transscleral cyclophotocoagulation (2 eyes). The enucleation decision was made based on the following indications: no light perception, uncontrollable IOP with more than four kinds of IOP lowering medications, patients refused intraocular anti-VEGF treatment or transscleral cyclophotocoagulation, and patients’ desire.

Forty-four (33.3%) patients suffered from tumor recurrence, 32 of them were accompanied by severe glaucoma. The median time to enucleation was 34.8 months (range: 12.5–110.8 months, IQR: 25.0–47.0 months) (Fig. 1). Thirty-four eyes had immediate secondary enucleation. Ten eyes had additional treatments for the attempt at globe salvage before secondary enucleation, of which nine received additional salvage TTT and one received TTT and second PRT.

Ten (7.6%) eyes received enucleation entirely due to other types of glaucoma. The median time to enucleation was 17.8 months (range: 4.3–102.0 months, IQR: 6.3–36.0 months) (Fig. 1).

Tumor non-response occurred in 18 (13.6%) cases, four of which were accompanied by severe glaucoma. The median interval from PRT to enucleation was 6.0 months (range: 0.5–11.9 months, IQR: 4.5–9.0 months). Two patients received additional salvage TTT, which failed to preserve their eyes.

Failure to preserve the eyes for other reasons occurred in eight (6.1%) patients. Six of them requested enucleation due to poor vision. One patient suffered from sclera necrosis. Endophthalmitis after intravitreal anti-VEGF treatment occurred in an eye with choroidal melanoma.

Pathology examination revealed some degree of tumor necrosis in all cases. These irradiated melanomas were of spindle type in 23 (17.4%) cases, epithelioid in 33 (25.0%) cases, and mixed in 36 (27.3%) cases. In 4 (3.0%) cases, both melanocytoma and melanoma cells were observed. Complete tumor necrosis precluded tissue diagnosis in 36 (27.3%) cases.

### Systemic outcomes and risk factors for metastasis and metastasis-related death

**Fig. 2:**
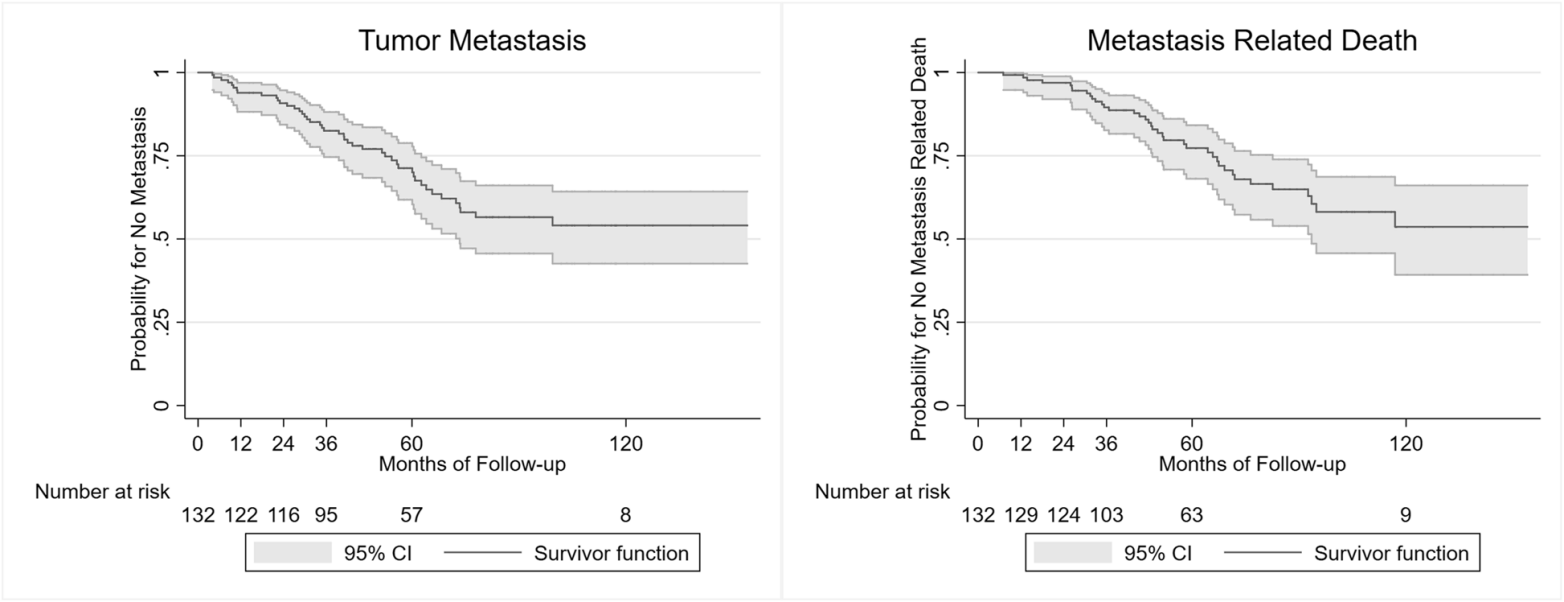
Kaplan–Meier analysis for patients free of metastasis and metastasis-related death with 95% confidence intervals

During follow-up, metastasis was detected in 45 (34.1%) patients, and 38 of them died. One patient died of stroke without metastasis. The 5-year metastasis rate was 28.7% (95% CI: 21.2%-38.2%) (Fig. 2). The median time from plaque implantation to metastasis was 39.5 (range 4.0–99.4, IQR: 22.9–60.1) months. The 5-year metastasis-related mortality was 22.7% (95% CI: 15.8–31.9%) (Fig. 2). The median time from plaque implantation to metastasis-related death was 48.7 (range 7.0–116.9, IQR: 32.0–67.5) months. The median time from metastasis to related death was 9.3 (range 3.0–54.0, IQR: 5.3–17.0) months.

Univariate and multivariate factors predictive of tumor metastasis and metastasis-related death are listed in Table 3.

**Table 3:** Analysis of risk factors of metastasis and metastasis-related death

	**UM Metastasis**		**Metastasis-related death**	
	**HRs (95%CI)**	***P* Value**	**HRs (95%CI)**	***P* Value**
**Univariable analysis**				
**Male**	1.25 [0.69, 2.24]	0.46	1.48 [0.78, 2.80]	0.23
**Age **^**a**^	1.33 [1.05,1.70]	**0.02**	1.53 [1.16, 2.01]	**0.002**
**BCVA**				
≥ 0.8	Reference		Reference	
< 0.1	0.85 [0.36, 2.00]	0.71	0.92 [0.35, 2.42]	0.87
0.1 ~ 0.29	0.89 [0.35, 2.27]	0.81	0.99 [0.35, 2.79]	> 0.9
0.3 ~ 0.49	0.60 [0.19–1.86]	0.38	0.61 [0.17, 2.18]	0.45
0.5 ~ 0.79	0.82 [0.28, 2.39]	0.72	1.06 [0.34, 3.29]	> 0.9
**Tumor Thickness**^**b**^	1.00 [0.89, 1.13]	> 0.9	1.03 [0.90, 1.17]	0.62
**LBD**^**b**^	1.12 [1.02, 1.23]	**0.017**	1.14 [1.03, 1.26]	**0.012**
**Tumor Anterior Margin**				
Equator to Macula	Reference		Reference	
Ora Serrata to Equator	1.95 [1.02, 3.76]	**0.044**	2.25 [1.09, 4.65]	**0.028**
Ciliary Body	1.93 [0.75, 4.97]	0.18	2.08 [0.73, 5.95]	0.17
**Tumor Posterior Margin**				
Macula	Reference		Reference	
Equator to Macula	0.53 [0.27, 1.02]	0.056	0.63 [0.31, 1.26]	0.19
Ora Serrata to Equator	0.40 [0.05, 2.98]	0.37	0.45 [0.06, 3.38]	0.44
**Subretinal Fluid**	1.40 [0.43, 4.52]	0.57	1.16 [0.36, 3.77]	0.81
**Optic Disc Involved**	0.68 [0.24, 1.91]	0.46	0.67 [0.21, 2.19]	0.51
**Vitreous Hemorrhage**	0.43 [0.06, 3.10]	0.40	0.56 [0.08, 4.11]	0.57
**Pathology (*n* = 92)**				
Spindle	Reference		Reference	
Mixed	2.12 [0.86, 5.28]	0.10	1.84 [0.70, 4.85]	0.22
Epithelial	1.98 [0.80, 4.87]	0.14	1.82 [0.72, 4.61]	0.21
Necrotic	0.80 [0.26, 2.46]	0.70	0.88 [0.27, 2.89]	0.83
**Local control failure**				
No response	4.78 [2.33, 9.79]	** < 0.001**	5.05 [2.43, 10.50]	** < 0.001**
Recurrence	1.97 [1.10, 3.54]	**0.023**	1.67 [0.88, 3.15]	0.12
**Complications**				
NVG	0.65 [0.36, 1.18]	0.16	0.71 [0.37, 1.34]	0.29
Other secondary glaucoma	0.63 [0.19, 2.02]	0.44	0.46 [0.11, 1.92]	0.29
**Multivariable analysis**				
**Age**^a^	1.26 [0.92,1.72]	0.15	1.54 [1.07, 2.23]	**0.022**
**LBD**^b^	1.15 [1.01, 1.31]	**0.035**	1.14 [0.98, 1.32]	0.079
**Local control failure**				
No response	7.22 [2.63, 19.82]	** < 0.001**	7.91 [2.79, 22.48]	** < 0.001**
Recurrence	3.29 [1.54, 7.07]	**0.002**	3.08 [1.13, 7.23]	**0.01**
**Tumor Anterior Margin**^c^	1.48 [0.69, 3.15]	0.31	1.67 [0.70, 4.00]	0.25

^a^Per 10-year increase

^b^Per 1-mm increase

^c^Equator to macula was the reference variable

Univariate analysis revealed that increased age at presentation (*p* = 0.02), tumor LBD (*p* = 0.017), tumor anterior margin (*p* = 0.044), tumor non-response (*p* < 0.001), and recurrence (*p* = 0.023) were associated with metastatic spread. On multivariate analysis, tumor LBD (*p* = 0.035), tumor non-response (*p* < 0.001), and recurrence (*p* = 0.002) remained significant factors.

Increased age (*p* = 0.002), tumor LBD (*p* = 0.012), tumor anterior margin (*p* = 0.028) and tumor non-response (*p* < 0.001) were risk factors for metastasis-related death on univariate analysis. On multivariate analysis, increased age (*p* = 0.022), tumor non-response (*p* < 0.001) and recurrence (*p* = 0.01) remained significant.

## Discussion

Brachytherapy has proved to be safe and effective for UM. Nevertheless, some patients underwent secondary enucleation owing to complications and poor tumor control. It is worth noting that, previously, only a few studies based on the Caucasian population focused on secondary enucleation cases after PRT. To our knowledge, there has been no similar large sample study on the East Asian population.

In 1989, Shields et al. [8] took tumor recurrence as the primary indication for eye removal for 59 UM patients after PRT, followed by NVG, patient request, scleral necrosis, painful bullous keratopathy, and hemolytic glaucoma. A similar study conducted by Fabian [16] et al. supported tumor recurrence as the most common reason for secondary enucleation. In the Collaborative Ocular Melanoma Study (COMS) trial [9], 69 eyes were enucleated during the first five years after brachytherapy, and treatment failure occurred in 57 eyes. The risk factors for secondary enucleation after PRT were increased tumor thickness, closer proximity of the posterior tumor border to the foveal avascular zone, and poorer baseline visual acuity [9].

In contrast to the past findings [8, 16], the current study showed that NVG was the most common indication for Chinese UM patients. In our study, 52 (39.4%) eyes were enucleated simply because of NVG. This proportion was much higher than those shown in previous studies. In Shields [8] et al.’s study, 59 patients were treated with various radioactive isotopes. The radiation dose to the tumor apex averaged 80 Gy, and the proportion of enucleation due to NVG was 31%. In Fabian [16] et al.’s study, the indication for secondary enucleation was NVG in 21%. In their study, 85 of 99 patients were treated by ^106^Ru plaque radiotherapy, and the median radiation dose to the tumor apex was 100 Gy. Higher tumor apical height [17], increased pack-years of smoking history [18], pseudophakia [18], and higher grade of radiation retinopathy severity [18] were risk factors for NVG. A higher radiation dose to adjacent tissue may induce a higher probability of NVG [19, 20], but whether it increases the incidence of NVG-related enucleation in the whole irradiated population is still unclear. Other modalities of radiation therapy(charged particle radiotherapy and stereotactic radiotherapy) also failed to escape from secondary glaucoma [21–23] and led to similar [23] even higher incidence [24, 25]. NVG, the main reason for secondary enucleation in our cohort, should be closely monitored throughout follow-up. Further research should be conducted on NVG prevention and treatment.

Intraocular anti-VEGF treatment is thought to be effective against NVG [26]. However, prophylactic intravitreal anti-VEGF treatment may have failed to prevent or delay the occurrence of either iris neovascularization (NVI) or NVG in irradiated eyes with UM [27]. In a retrospective and nonrandomized cohort study [27], 1131 eyes that received repeated prophylactic bevacizumab (1.25 mg in 0.05 mL each time) were compared with 117 eyes that didn’t. No difference was identified between the two groups in the incidence or mean time to develop NVI or NVG. However, this study has two limitations. First, the first dose was given at plaque removal, and the radiation had caused ischemia. Second, NVI and NVG severity differences were not mentioned. Thus, we can’t entirely exclude the effectiveness of prophylactic anti-VEGF treatment. The therapeutic effect, occasion, and dose are still worth being studied.

Tumor recurrence was the second reason, leading to 33.3% of secondary enucleation. Nearly 90% of the recurrences occurred within five years after PRT. Still, three patients eventually lost their eyes nine years after the initial treatment. This emphasizes the necessity of long-term regular ocular and systemic follow-up for UM patients after eye salvage treatments. In addition, 18 patients in this study were insensitive to radiotherapy. The tumor did not shrink and even grew within one year after treatment. A previous study suggested that juxtapapillary tumor was one of the risk factors for tumor non-response [16]. However, only one juxtapapillary tumor showed non-response in our study.

The 5-years metastasis rate for the whole cohort was 28.7%, and metastasis-related mortality was 22.7%, which were higher than the figures in previous research [4, 28–30]. This may be due to selection bias as we only included secondary enucleation cases. Our results showed that tumor non-response and recurrence increased the risk of metastasis and metastasis-related death, corresponding to prior work [3, 16, 31, 32]. We speculate that the radiation-resistant tumors may be more invasive and easier to spread. Therefore, it is essential to pay more attention to the systemic condition and increase the follow-up frequency, if necessary, for the non-response and recurrence cases.

To our knowledge, our study is the first on enucleated UM patients after PRT in East Asians with a large sample and detailed follow-up. This study directs complications control as a significant research direction and contributes to outcome prediction and indications selection. Subsequently, patients with high-risk factors can have personalized observation, including individualized follow-up intervals and examination items. However, this study is limited by the nature of the retrospective study and the lack of unenucleated cases. There is also a single-center bias in our study, as we excluded patients operated in other hospitals. Furthermore, molecular genetic analysis was unavailable in this research as it was not routinely available in our practice at the time of treatment. Despite the limitations, our study contributes to the current literature on enucleation after PRT for its analysis of East Asian cases with comprehensive data.

In conclusion, in this 12-year study, NVG was the prime reason for eye removal for Chinese UM patients after brachytherapy. Tumor non-response and recurrence increased the risk of metastasis and related death.

## Data Availability

The original data and material of the current study are available from the corresponding author upon reasonable request.
